# Cavernous tissue preservation technique versus conventional technique during penile prosthesis implantation: a prospective comparative study

**DOI:** 10.1007/s00345-025-05476-w

**Published:** 2025-02-24

**Authors:** Mohamed Abdelrahman Alhefnawy, Hazem Abdelsabour Deif, Ahmed Farag wahsh, Mohamed Gamal Ahmed, Ahmed Mohammed El-Taher, Gamal Abdelmalek Morsy, Alaa Rafaat Mahmoud, Helmy Ahmed Eldib

**Affiliations:** 1 Urology Department, Benha University, Benha, Qalubia Egypt; 2 Urology Department, Al-Azhar University, Asyut, Asyut Governorate Egypt; 3 Urology Department, Asyut University, Asyut, Asyut Governorate Egypt

**Keywords:** Cavernous tissue, Penile prosthesis, Penile tumescence, Erectile dysfunction, Penis

## Abstract

**Background:**

Few prospective studies in literature with long postoperative follow-up compared between cavernous tissue sparing and conventional penile prosthesis implantation techniques.

**Aim:**

To compare between cavernous tissue sparing and conventional penile prosthesis implantation techniques in terms of patient and partner satisfaction and perioperative outcomes.

**Methods:**

In All, 60 Patients with severe erectile dysfunction were randomized into 2 equal groups; patients undergoing conventional malleable penile prosthesis implantation, and patients undergoing the cavernous tissue-sparing technique. Postoperatively, prosthesis function and patient satisfaction were assessed at 6 weeks after surgery and then 3–6 and 12 months using EDITS and QoLSPP questionnaires. Patients were asked about residual penile tumescence. Perioperative data were recorded.

**Results:**

Modified EDITS questionnaire after 3,6, and 12 months was 76.9 ± 18, 79 ± 17 and 82.3 ± 16 respectively. As QOLSPP questionnaire, 46 (73.8%) subjects were highly satisfied, 25 patients (83.3%) in cavernous tissue sparing and 21 patients (70.00%) in Conventional group. While 14 (26.2%) were less satisfied, 5 patients (16.7%) Cavernous tissue sparing and 9 patients (30.00%) in Conventional group. In the cavernous tissue-sparing group, 26 of 30 patients (86.6%) reported having a significantly higher incidence of residual penile tumescence versus 2 of 30 patients (6.6%) in the conventional surgery group (P < .001). The age of highly satisfied subjects was significantly lower than those less satisfied (p = 0.025), while the BMI of highly satisfied subjects was significantly lower than those less satisfied (p = 0.001).

**Conclusion:**

There is a significantly higher incidence of residual penile tumescence in Cavernous tissue sparing group. Many factors affect male satisfaction rates after PPI as age, and BMI.

**Supplementary Information:**

The online version contains supplementary material available at 10.1007/s00345-025-05476-w.

## Introduction

Erectile dysfunction is a major health problem for sexual active men because of the impairment in quality of life (QoL). The common causes of erectile dysfunction include Organic (neurogenic, vasculogenic, endocrinologic), psychogenic reasons, Chronic systemic diseases like Diabetes mellitus and radical prostatectomy [1].

Treatment options for ED are oral pharmacotherapy, vacuum erection devices, intracavernosal injections, intrauretheral implant and penile prosthesis [2].

The implantation of penile prosthesis is an effective option for treating erectile dysfunction (ED) that is used to treat those cases not responsive to approved medical treatment. Several types of prosthesis have been developed since their introduction in 1970s, and the most common devices implanted today are represented by two-piece prosthesis [3].

Malleable prosthesis permits penile rigidity and flaccidity and so have a better cosmetic result [4]. The types of prosthesis most commonly implanted are the three-piece inflatable device, the two-piece inflatable device, and the soft and malleable prosthesis, which are reliable and inexpensive [5].

Antibiotic coverage, surgical and device improvement have also changed, resulting in a safer procedure and better longevity of device with consequent high level of patient and partner satisfaction [6]. The design and placement techniques of this kind of device have dramatically improved and nowadays most complications are related to medical aspects rather than failure of the prosthesis, despite the widespread use of penile prostheses, knowledge of the psychological and interpersonal impacts of these implants remains limited [7].

Experts feel that personal dissatisfaction with a penile implant procedure is more common, and is usually due to unreasonable or inappropriate expectations for the procedure or more extensive destruction of corporal tissue that reduce erectile function post implantation of penile prosthesis [8]. The cavernous tissue sparing technique has the adavantage of preserving the ability of the penis to enhance the tumescence and penile girth with no significant perioperative complications [9].

Therefore, in our study we compare between cavernous tissue sparing technique and the more destructive conventional technique.

## Patients and methods

Between May 2019 and May 2022 sixty patients, admitted to the Urology Department, presenting with severe organic ED (SHIM < 7) for at least one year, refractory psychogenic ED and ED not responsive to approved medical treatment were randomized into 2 equal groups; patients undergoing conventional malleable penile prosthesis implantation, and patients undergoing the cavernous tissue-sparing technique in order to compare between both groups in terms of postoperative patient and partner satisfaction and perioperative outcomes. Diagnosis was set by SHIM questionnaire [10], ICI test and penile color duplex scanning. Exclusion criteria were Patients’ age ≥ 70 years and ≤ 20 years, Patients with end organ failure or compromised immune system, Patients with acute urinary tract infection or uncorrectable coagulopathy, Patient with uncontrolled DM and/or high HbA1c > 7.5 and Patients with previous penile prosthesis surgery. An informed consent was obtained from all patients and this study was conducted according to ethical principles stated in the Declaration of Helsinki (2013) [11] and the requirement of faculty of medicine, Al-Azhar university. Preoperative laboratory investigations included complete blood cell count (CBC), coagulation profile, Random blood sugar, HBA1C, Lipid profile, liver function tests serum testosterone, serum prolactin and urine analysis &culture.

Patients’ demographic data, ED characters, risk factors, SHIM score data, ICI diagnostic test data, laboratory tests data, penile duplex interpretation, perioperative data: (Operation time (min), Incision, Penile girth (pre&postoperative),complications (Intra&Postoperative), prosthesis function and patient satisfaction at 6 weeks after surgery and then 3–6 and 12 months using EDITS and QoLSPP questionnaires were recorded.

### Operative technique

Patients were classified into two equal groups according to technique of operation: Group I: conventional malleable penile prosthesis insertion group, Group II: cavernous tissue–sparing group. The Non-Touch Technique was designed to reduce penile prosthesis infection by markedly limiting contact with the patient’s skin.

**Fig. 1:**
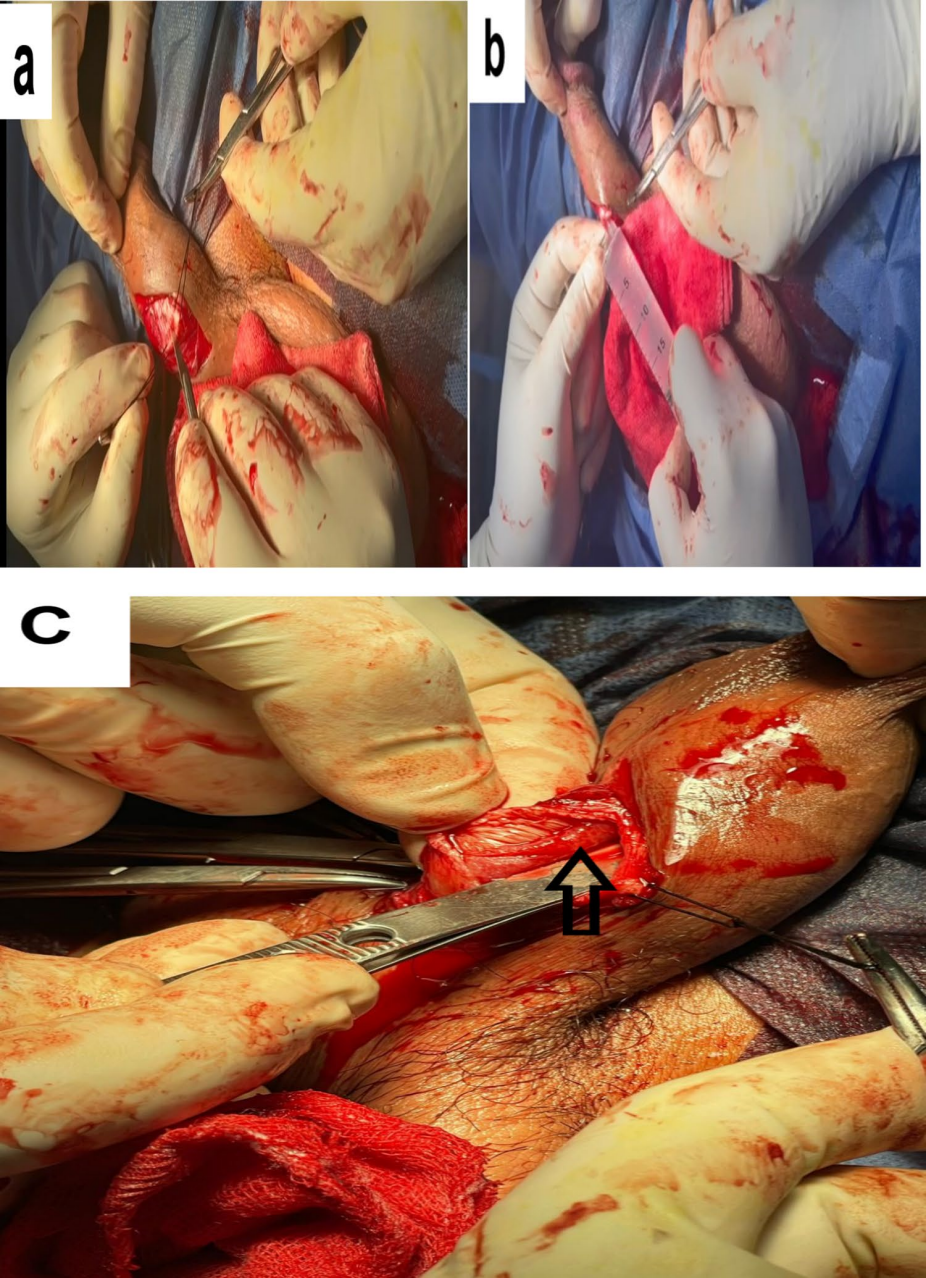
**a**: Placing two stay sutures in the tunica albuginea. A small incision was made on the corpora cavernosa to expose the corporeal spongy tissue **b**: Hydrodissection by 20 cm saline syringe to create a plane between tunica albuginea and corpus cavernosum. **c** Preserved corpus cavernosum tissue as shown by arrow

A 3 cm incision was made at the penoscrotal junction at the median raphe and carried down to the superficial/dartos fascia. The corpora cavernosa were exposed on each side then we placed two stay sutures in the tunica albuginea (Fig. 1a). A small incision was made on the corpora cavernosa to expose the corporeal spongy tissue (Fig. 1a). In the conventional group applying standard procedure, the next step was to dilate the corpus cavernosum spongy tissue with a blunt Hegar dilator, insert10-mm Hagar's dilator until it fitted well beneath the glans. Progress through larger dilators, pointing the curve antero-laterally, to 12 or even 14 mm.

While in cavernous tissue-sparing group we used hydro dissection by 20 cm saline syringe to create a plane between tunica albuginea and corpus cavernosum (Fig. 1b) followed by dilatation solely with 8 size dilator to 10 sized dilator in order to be able to preserve the corpus cavernosum tissue (Fig. 1c).

We inserted dilator proximally then we used scissors in cases of corporeal fibrosis to find the right way into the corpora taking care not to perforate the crus. We stopped when the dilator is held up at the ischial tuberosity. We Changed gloves before handling the prosthesis, measured the length needed for the prosthesis by metal measuring tool 1–2 cm less, the proximal end of the prosthesis was inserted first, and then we bent the distal end into a loop or circle with the aid of the assistant to slip it into the corpus. If it was too short and the glans drooped downward, we added appropriate rear tip extenders. We irrigated the wound with garamycin solution. We performed the same procedure on the other side. We closed the tunica albuginea with a running 2–0 vicryl, checked the position of the paired prosthesis and irrigated the wound again. Lastly, we closed the subcutaneous tissue and skin with 2–0 vicryl.

We measured the penile girth mid-shaft preoperative and postoperative, and the length was measured from the symphysis pubis to the tip of the glans in both relaxed and stretched states.

Postoperatively, broad-spectrum antibiotic IV injection was given for 5 days in the form of ceftriaxone and metronidazole then tablets in the form of quinolones and amoxicillin-Clavulinic to prevent infection for 10 days [12]. The Foley catheter was left in for one day. Each patient was followed at the outpatient clinic until the surgical wound healed, and surgical complications were recorded in detail.

Data was analyzed using Numerical data were explored for normality by checking the distribution of data and using tests of normality (Kolmogorov–Smirnov and Shapiro–Wilk tests). SPSS version 23.0 was used for data management and data analysis. median and range when appropriate described quantitative data. Student t-test was used to compare data if normally distributed, while Mann–Whitney U test and Wilcoxon Signed Ranks Test were used if the data are not normally distributed. Qualitative data were presented as frequencies and percentages, Chi square test and Fisher Exact test were used for comparison between the 2 groups. The significance level was set at P ≤ 0.05. Statistical analysis was performed with IBM SPSS Statistics for Windows, Version 23.0. Armonk, NY: IBM Corp.

## Results

80 patients were recruited into the study, 7 patients dropped out of the study, 5 patients were lost to follow-up and 8 patients had incomplete data file.

**Fig. 2:**
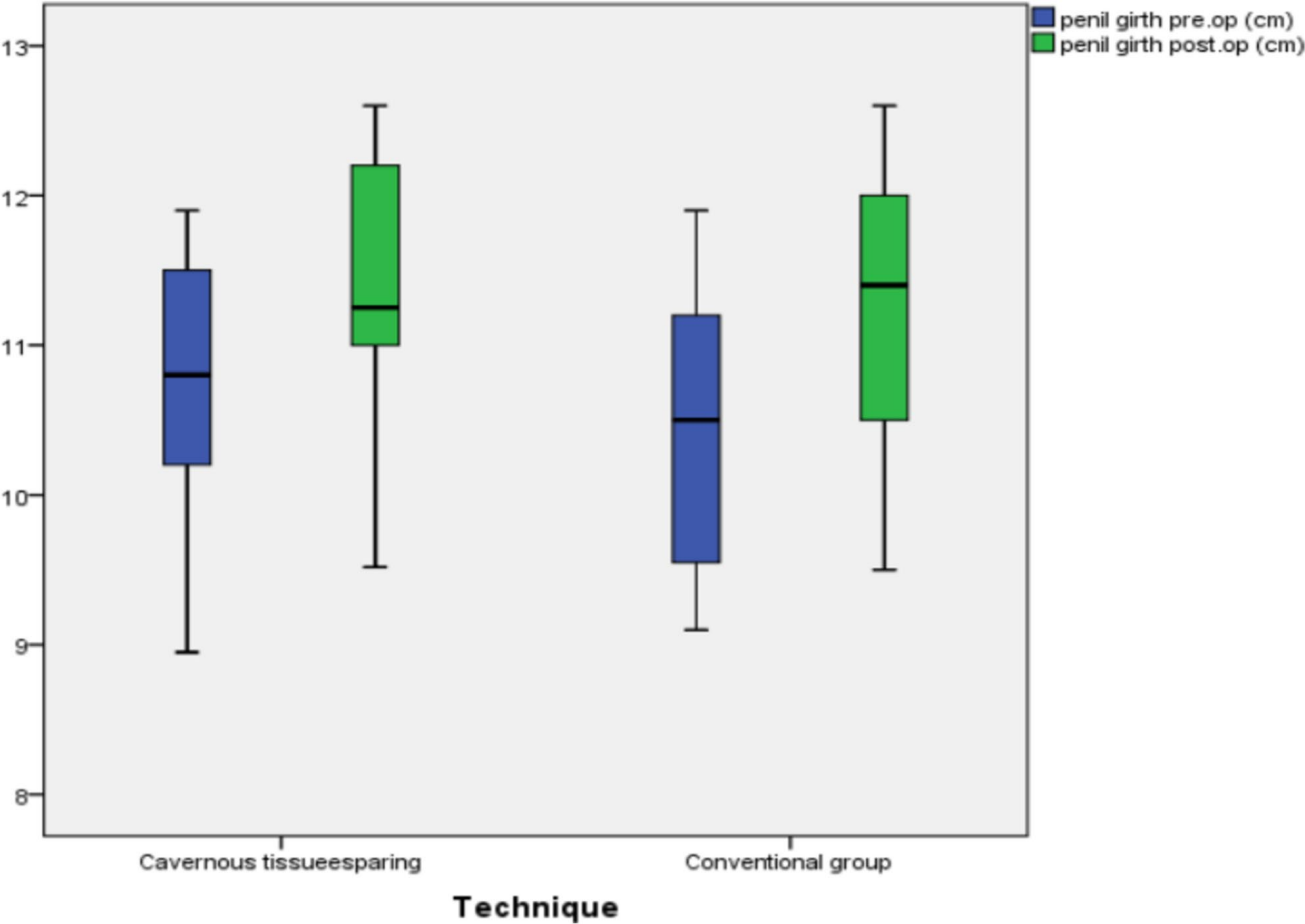
Box and whisker plot showing penile girth (cm) pre and post-operative between two studied techniques

In all, 60 cases were eligible for the study aging 30–67 years with a median age of 55 years were included in this study. The risk factor and past history in our study group (Table 1) showed that the majority of patients had a hypertension 35 (58.3%), diabetes 34 (56.7%) and most of them were smokers 36 (60%). Also, only16 (26.7%) of patients had chronic heart disease, 5 patients (8.3%) had Peyronie’s disease, 7 patients (11.7%) had history of trauma and 7 patients (11.7%) had past history for surgery (3 cases with fracture penis, 3 cases with history of radical cystectomy and one case with radical prostatectomy). Operative time ranged (50–120) minutes, mean time was (60) minutes, operative time was prolonged mainly with Peyronie’s disease that can reach to 120 min. the difference in comparison with other risk factor statistically significant (P value = 0.001). As regard relation of QoLSPP to risk factor in two study group, there was no significant difference between the conventional group in terms of HTN, DM, CHD, surgery, and penile duplex interpretation (p = 0.118, 0.419, 0.195, 1.000, and 1.000, respectively).There was, however, a significant relationship between the conventional group, age, and BMI. The age of highly satisfied subjects was significantly lower than those less satisfied (p = 0.025), The BMI of highly satisfied subjects was significantly lower than those less satisfied (p = 0.001). Demographics and perioperative data were as shown in Tables 1, 2 respectively. Penile girth pre and post PPI and Post-operative patient’s satisfaction by modified EDITS and QoLSPP questionnaires and Spontaneous Penile Tumescence were as shown in Tables 3, 4 respectively (Fig. 2).

**Table 1:**
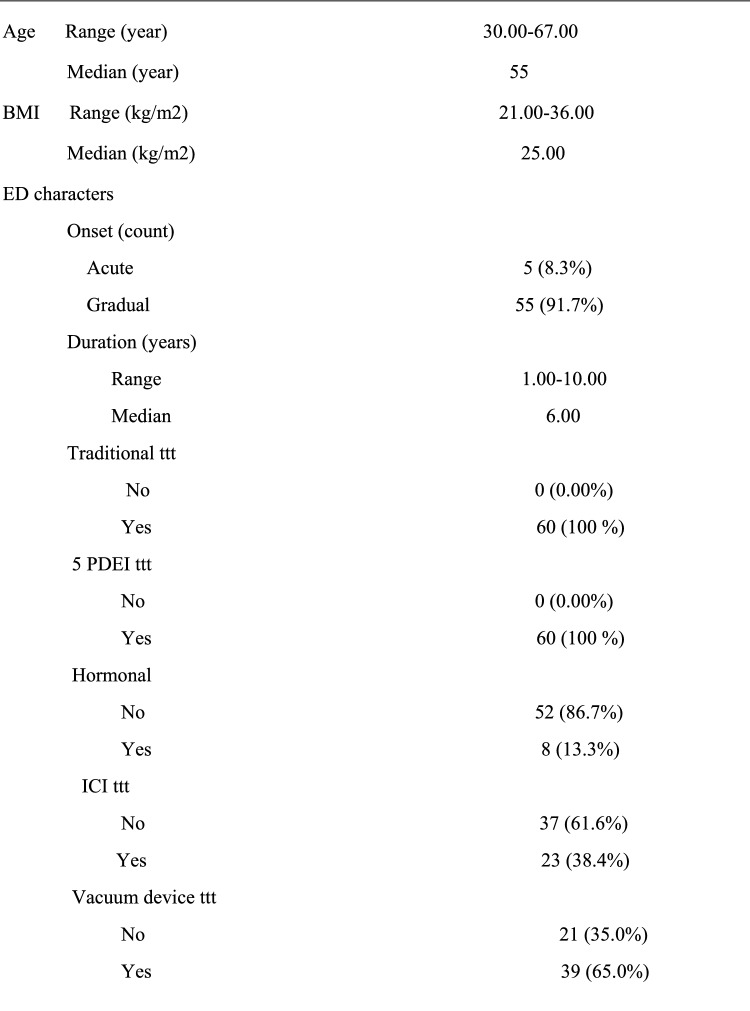
Baseline characteristics

**Table 2:**
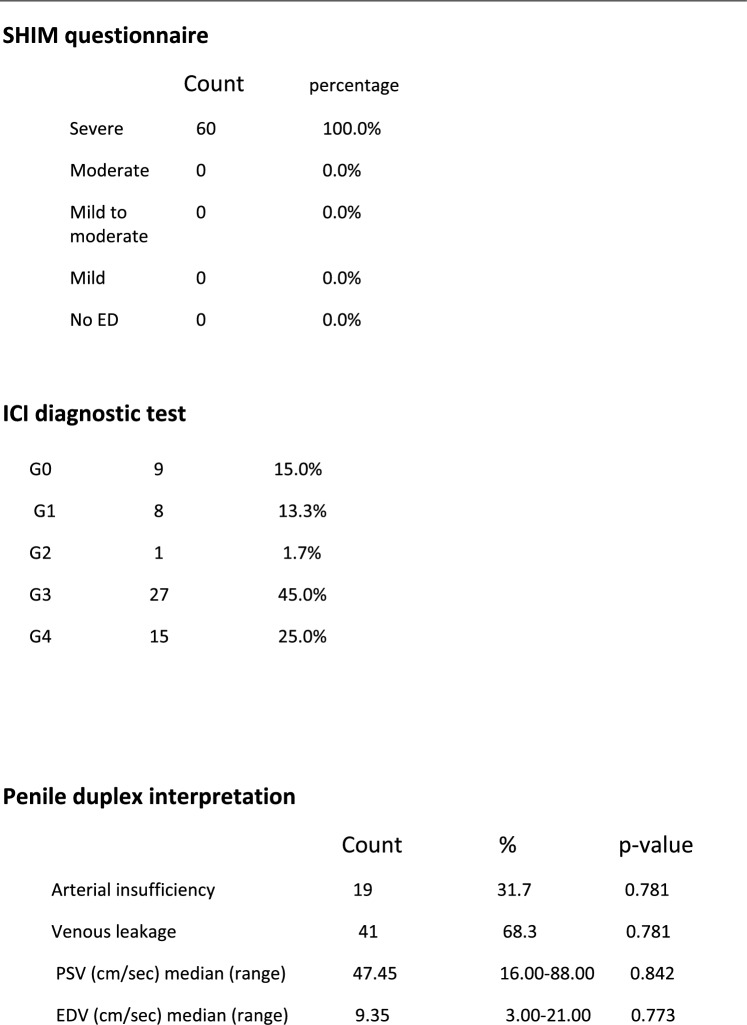

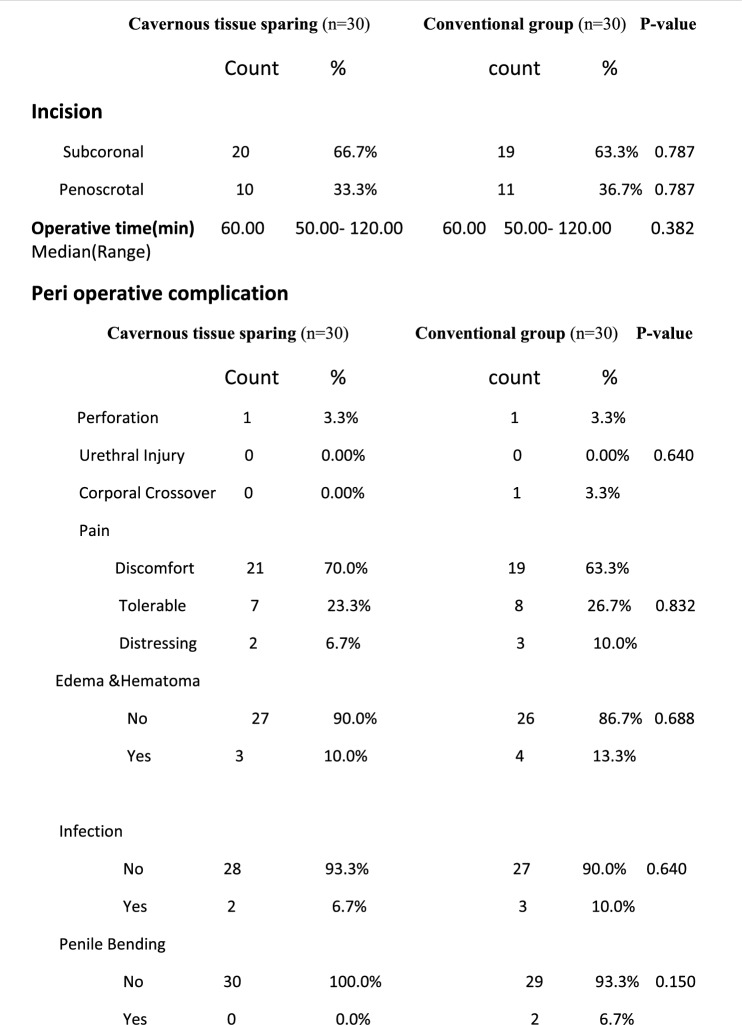
Perioperative data

**Table 3:** Penile girth pre and post PPI

	Study group (n = 60)						p-value
	Cavernous tissue sparing (n = 30)			Conventional group (n = 30)			
	Median	Range		Median	Range		
Penile girth pre-op (cm)	10.80	8.95	11.90	10.50	9.10	11.90	0.346
Post-op (cm)	11.25	9.52	12.60	11.40	9.50	12.60	0.453
p-value over time	< 0.001*			< 0.001*			

Bold values indicates penile girth is significant in both groups with p-value < 0.001

*Significant at P ≤ 0.05

**Table 4:** Post-operative patient’s satisfaction by modified EDITS and QoLSPP questionnaires and Spontaneous Penile Tumescence

		Study Group (n = 60)				P-value
		Cavernous tissue sparing (n = 30)		Conventional group (n = 30)		
		Count	%	Count	%	
EDITS at 3 months interpretation	Very unsatisfied	1	3.3%	1	3.3%	0.848
	moderate unsatisfied	9	30.0%	12	40.0%	
	Moderate satisfied	19	63.3%	15	50.0%	
	satisfied	1	3.3%	1	3.3%	
	Very satisfied	0	0.0%	1	3.3%	
EDITS at 6 months interpretation	Very unsatisfied	0	0.0%	0	0.0%	0.729
	moderate unsatisfied	1	3.3%	2	6.7%	
	Moderate satisfied	7	23.3%	10	33.3%	
	satisfied	14	46.6%	12	40.00%	
	Very satisfied	8	26.6%	6	20.00%	
EDITS at 12 months interpretation QoLSPP after 6 months	Very unsatisfied	0	0.0%	0	0.0%	
	moderate unsatisfied	0	0.0%	0	0.0%	0.207
	Moderate satisfied	1	3.3%	3	10.0%	
	satisfied	4	13.3%	8	26.7%	
	Very satisfied	25	83.3%	19	63.3%	
	Less satisfaction	5	16.7%	9	30.0%	0.222
	High satisfaction	25	83.3%	21	70.0%	0.222
Residual Spontaneous Penile Tumescence		26	86.6%	2	6.6%	< .001*

## Discussion

Erectile dysfunction, defined as the inability to attain or/and maintain an erection sufficient for satisfactory sexual performance[13].The most important end-point of PPI surgery is to achieve the highest patient and partner satisfaction with the lowest complication rates[14]. Although the satisfaction rates have been well studied, the factors affecting the satisfaction levels post PPI have not been well-studied [15].

To our knowledge, few prospective studies compare between conventional and cavernous sparing techniques in PPI. Therefore, in this study we aimed to compare between both techniques along with analyzing the factors affecting male satisfaction rates after PPI.

Our study is a prospective clinical study included 60 patients, with severe ED. In this series, median age was 55 years, with a range of (30–67). While In a study done by Chung et al.(2013) [16], the median age for penile prosthesis implant was 53 years (range from 28 to 80 ys), and in another study done by Dhabuwala et al. (2011) [17], the median age for penile prosthesis implant was 61 years (range from 35 to 85 ys). In both studies, there is significant difference in the maximum age in comparison with our study mainly due to decrease in life expectancy in our developing country, which is below (75 y). The duration of ED before surgery ranged from (1–10) years with a median of 6 years which is similar to study done by Ibrahim et al. (2015) [18], that reported a median duration of 6.6 years. In our study, we did not observe an association between the duration of ED before PPI and the male satisfaction rates post PPI that is in agreement with others [18].

In our study there were multiple risk factors and etiology of ED the most prevalent of them was smoking, 36 patients (60.0%), this agrees with study done by RM Seyam et al. (2017) [19] in which ED was directly associated with smoking cigarettes or tobacco (P > 0.005). Long- term cigarette smoking is a major risk factor for development of vasculogenic ED because of its effects on the vascular endothelium and the prevalence of severe ED among heavy smokers is 19.6% [20].

In our study all patients experienced medical treatment in the form of oral sildenafil and (38.4%) of patients had home ICI therapy after failure of oral sildenafil treatment but also failed, while vacuum device was used by the fewest number of patients, which is consistent with the data obtained from study done by Salama et al. (2004) [21]. While in the study done by Nguyen HMT et al. (2017) [22], (52.5%) of total 139 patients had used oral medication for ED before surgery, and (31.7%) of total 139 patients had used ICI injections.

The Sexual Health Inventory for Men (SHIM) is a widely used scale for screening and diagnosis of erectile dysfunction (ED) and severity of ED in clinical practice and research. Using SHIM score criteria all patients (100%) in this study were ≤ 6 and mean score was 5 as all patients subjected to the implant surgery had severe ED, similar to result of study done by others [23] with a mean score preoperatively of 5.2.

As regard ICI diagnostic test sixty patients has been subjected to ICI test, ICI response was ≤ 3, and reached grade 4 only in 15 patients who had venous leakage with very rapid detumescence, similar to result of study done by other authors [24] in which up to 20% of patients with normal response (full rigid erection) were affected by arterial insufficiency as showed by penile Doppler ultrasound studies.

According to the results of the penile duplex obtained in our study, all patients (100%) were diagnosed as having vasculogenic ED (41 patients venogenic ED and 19 patients with arteriogenic ED) in comparing with the data base obtained from Song WD, et al. (2013) [25], 70% of patients are vasculogenic and with the study done in china by others [26] on 40 patients, there were 35 cases with neurogenic ED, the rest included five cases with venous leakage. This explains the concept of penile prosthesis that was accepted in patients with neurogenic ED in the previous study, as most of the patients were neurogenic.

In our study, operative time ranged (50–120) minutes, mean time was (60) minutes, operative time was prolonged mainly with Peyronie’s disease, ranged (90–120 min), and the difference in comparison with other risk factor was statistically significant (P value = 0.001). This can be explained by slow and careful blunt dilatation to expand the corpora cavernosa via standard corporotomy incision in order to avoid undesirable complication [27], while in another study [18], the mean operation time was 46.6 ± 10.9 in malleable penile prostheses group.

In this study, there was no significant difference in the preoperative median stretched penile girth between the 2 groups (10.80 vs 10.50 cm), while the postoperative mean penile girth was significantly improved in both study groups from pre-operative, 11.25 cm with range (9.50–12.60) cm, (p =  < 0.001), similar to result of another study [28]**,** the postoperative mean penile girth was significantly thicker post penile prosthesis insertion (9.79 ± 1.11 cm). This girth preservation renders the penis more aesthetic and makes it more natural after surgery. This point could potentially decrease the demand for further postoperative girth-enhancing procedures often requested by patients by undergoing either silicone implantation or fat injection.

Regarding satisfaction post-operative with different operative steps during penile prosthesis implantation Conventional group or cavernous tissue sparing, there was no significant difference between two groups in male satisfaction rates post PPI, and this differ with results that reported by other authors[29], due to decrease in sample size in this study.

In our study, there was significant difference between two groups concerning spontaneous penile tumescence, In the cavernous tissue-sparing group, 26 of 30 patients (86.6%) reported having a significantly higher incidence of residual penile tumescence versus 2 of 30 patients (6.6%) in the conventional surgery group (P < 0.001) which is in agreement with the results of the study by Zaazaa A, et al. (2019) [28].

Intra-operatively we detected one case of distal Crossover, and two cases of perforation, and these complications only occurred with patients with Peyronie’s disease. We managed these complications intra-operatively successfully these results were comparable to the study by others[18] in which there were corporeal crossover (5 in MPP, 2 in IPP), corporeal perforation (1 in MPP, 3 in IPP) and urethral perforation (1 in MPP,1 in IPP). As regard post-operative superficial infection occurred in 5 patients of total 60 (8.3%), and managed successfully, three patients with superficial infection were diabetic, and may be also referred to lack of personal hygiene of the patients, ignorance to post-operative instruction as antibiotic dosage, early sexual intercourse in the 1st couple weeks. The incidence of infection was relatively higher than the data base obtained by some authors [29]**,** in which infection occurred in 8 patients of total 100 (8%), while in a study done by other authors [17] from 81 patients, 8 patients developed infection (4.4%) two of the eight patients were diabetic and one of these two was also on corticosteroid therapy for control of thrombocytopenia. Hematoma is another complication associated with an increased risk of infection. It usually presents in the early postoperative period, with an incidence ranging from 0.2 to 3.6%) [30]. Regarding Post-operative oedema & hematoma detected in this study 7 patient, three patients had DM and peyronie’s, two had peyronie’s disease only, all cases managed conservatively without operative intervention, while in another study [31], only one case (0.8%) hematoma developed postoperatively, requiring operative evacuation, while in another study[32] hematoma developed in 2(1.1%) cases and treated conservatively.

As regard penile floppy glans during intercourse occurred only in 2 patients, these nearly the same in comparison with the study done by other authors [32] in which penile penile floppy glans during intercourse was reported in 4(2.2%) patients.

Erectile Dysfunction Inventory of Treatment Satisfaction (EDITS) Questionnaire Patient satisfaction was evaluated using modified EDITS, which is a standardized assessment tool adapted to PP devices. EDITS is a validated questionnaire developed by Althof, et al. (2000) [33] to assess satisfaction following medical ED treatment.

In this study, there is gradual increase in patients’ satisfaction by EDITS from 3 months, 6 months and 12 months in two groups. In Conventional group Very satisfied patient at 3 months 1 patient (3.3%) while in 6 months 6 patients (20%) while in 12 months 19 patients (63.3%). while in cavernous tissue sparing group Very satisfied patient at 3 months 0 patient (0.00%) while in 6 months 8patients (26.6%) while in 12 months 25 patients (83.3%). So, there is no significant difference between the two groups in postoperative satisfaction as measured by the EDITS questionnaire at 3, 6, and 12 months (p = 0.848, 0.733, and 1.000, respectively).

While post-operative mean EDITS score using the Modified Erectile Dysfunction Inventory of Treatment Satisfaction (EDITS) questionnaire after 3,6, and 12 months was 76.9 ± 18, 79 ± 17 and 82.3 ± 16 respectively. while in another study [34], mean EDTIS score at 3,6, and 12 months was 58 ± 11,63 ± 9, and 81 ± 7 respectively, in comparing with the data base reported by some authors [35],overall mean EDTIS score being 77.1% and 75.6% for Genesis and Spectra malleable prostheses respectively, also using the EDITS questionnaire for patients with Spectra malleable prosthetic implants and their partners, another study [36], found a satisfaction rate of 86.4% in patients. The use of either a non-validated questionnaire or a tool developed in a non-PPI population could lead to an improper estimation of patient satisfaction. Indeed, with both strategies, we could miss the evaluation of some relevant aspects dealing with QoL, such as the relationship with the partner, as well as functional aspects, such as device operability and simplicity of use, which are all relevant to penile prosthesis surgery [37].

To provide a reliable tool able to simultaneously evaluate perceived penile prosthesis function and postoperative QoL, Caraceni and Utizi (2014) [38] designed the QoLSPP questionnaire, a validated questionnaire that specifically examined patient’s quality of life after PPI and the extent to which a penile prosthesis affects the patient’s sexual quality of life (SQoL). The main outcome measure of the QoLSPP is quality of life as biological and psychosocial-relational well-being after penile prosthesis placement. It has 16 questions encompassing 4 domains investigating: prosthesis function (functional); relationship with partner (relational); relation to the outside world (social); and self-image (personal) [38].

In this study, according to satisfaction using Quality of Life and Sexuality with Penile Prosthesis (QoLSPP) questionnaire after 6 months reported that high satisfaction 47 patients (78.3%) patients and Less satisfaction in 13 patients (21.6%), similar to study done by others[14] found some patients with low levels of QoL (10 patients with mean item scores < 2, 14.9%). On the other hand, 85.1% of patients reported high levels of satisfaction for the QoLSPP item regarding prosthesis function. This distribution might be due to some patients still having mental anguish connected to the feeling of impotence even though the prosthesis function is fine.

Regarding to factors affecting male satisfaction rates after PPI in our study the median age of highly satisfied subjects was 55.00 years, with range (30.00–65.00), while the median age of less satisfied subjects was 58.00 years, with range (45.00–67.00). The age of highly satisfied subjects was significantly lower than those less satisfied (p < 0.010).

So that, in this study the age of the patient has an effect on his satisfaction rates post PPI, thus confirming studies that have reported that they have observe an association between age and patient satisfaction, even after using a questionnaire specifically investigating the simplicity of use of the implanted device[39].

In another study [40], there were 45 patients (64%) who were younger than 65 years and all of them reported to be almost or very satisfied by the 2-piece IPP function, belonging the low rate of dissatisfaction only to patients older than 65 years, which could be explained by the lower expectations among elder patients.

Regarding to other factors affecting male satisfaction rates after PPI other authors [19] reported that a BMI > 30 kg/m^2^ has been associated with dissatisfaction after penile prosthesis surgery. This also has been shown through lower EDITS scores in this group compared with the general population, similar to results in this study, there is a positive relationship between BMI and satisfaction. Patients with high satisfaction BMI ≤ 24.5 kg/m^2^, while less satisfaction BMI > 30 kg/m^2^, so The BMI of highly satisfied subjects was significantly lower than those less satisfied (p < 0.001). Some data indicate that obese patients have a higher risk for ED than those with a normal BMI (≤ 25 kg/m^2^) because of abnormal endothelial function and psychological factors Thus, obese patients might harbor lower satisfaction at baseline before penile implantation that could explain lower postoperative satisfaction [41].

Regarding to risk factors we observed that 34 diabetic subjects, 23 (51.1%) subjects were highly satisfied and 11 (31.7%) were less satisfied complications on male satisfaction rates post PPI, significant difference was found between highly satisfied and less satisfied subjects regarding the frequency of DM (p < 0.022). These results are in agreement with the results of another study [39].

Regarding to etiology of ED and relation of this with satisfaction, 19 subjects with arterial insufficiency, 15 (31.9%) subjects were highly satisfied and 4 (30.8%) were less satisfied. While 41 subjects with venous leakage 32 (68.1%) subjects were highly satisfied and 9 (69.2%) were less satisfied. No significant difference was found between highly satisfied and less satisfied subjects regarding the frequency of arterial insufficiency or venous leakage (p = 0.937). These results are in agreement with the results of the study by other authors [39].

Our study has limitations: the small sample size and usage of only one type of penile prostheses (malleable) due to the financial aspect of implant surgery; these may be the subject of other researches to avoid these points and limitations.

## Conclusion

Cavernous sparing penile prosthesis implant provide comparable outcomes to conventional technique with no significant difference in perioperative complications with the benefit of preserving residual erectile function and residual postoperative penile tumescence.

Many factors affect male satisfaction rates after PPI as age, and BMI. The age of highly satisfied subjects was significantly lower than those less satisfied, while the BMI of highly satisfied subjects was significantly lower than those less satisfied.

## Supplementary Information

Below is the link to the electronic supplementary material.

Supplementary file1 (XLSX 45 KB)

## Data Availability

Sequence data that support the findings of this study data is available and provided within supplementary material files.
